# Seismic Performance of Steel-Reinforced Concrete Columns with Q690 High-Strength Steel

**DOI:** 10.3390/ma15092979

**Published:** 2022-04-19

**Authors:** Jun Wang, Xinyu Yi, Qi Liu, Xueqi Fang

**Affiliations:** School of Civil Engineering, Northeast Forestry University, Harbin 150000, China; liu_q_wangyi@163.com (Q.L.); fangxueqi@163.com (X.F.)

**Keywords:** high-strength steel, steel-reinforced concrete frame column, low-cycle loading tests, seismic performance

## Abstract

In this paper, based on the low-cycle loading tests of 11 steel-reinforced concrete (SRC) frame columns with built-in Q690 steel and 5 SRC frame columns with built-in Q235 steel, a systematic study on their seismic performance was carried out. The design parameters of the specimens were the steel strength, axial compression ratio, shear span ratio, steel content, and stirrup ratio. The failure modes, stress characteristics, hysteresis curve, skeleton curve, displacement ductility performance, energy dissipation capacity, and other main seismic indicators of the specimens with different parameters were analyzed, and the corresponding relationship between the displacement ductility performance of the specimen and the energy dissipation capacity and design parameters was obtained. The results show that the load–displacement curve of the specimens is relatively full, the descending section is gentle, and various seismic performance indicators are relatively excellent, reflecting good seismic performance. Equipped with high-strength steel SRC frame columns, they can better bear the horizontal load, the displacement ductility performance is improved, and the energy dissipation capacity is slightly lower than that of ordinary-strength steel SRC frame columns. The increase in the shear span ratio, steel content, and stirrup ratio of the specimens helps to improve their seismic performance, whereas an increase in the axial compression ratio makes their seismic performance worse.

## 1. Introduction

In recent years, with the advancement of metallurgical production technology, high-strength steels such as Q460 and Q690 have been successively developed and produced, and have also been successfully used in landmark buildings such as the Bird’s Nest, the Water Cube, and the Sony Center Building in Berlin, Germany [1]. High-strength steel has excellent mechanical properties, which can improve the mechanical properties of structures [2,3,4,5]. Aiming at the mechanical properties of high-strength steel, a calculation method based on density functional theory was proposed from the atomic level, effectively revealing the mechanism of metal strength and providing guidance for industrial production [6,7]. In order to meet the rapid development of modern high-rise buildings, it is necessary to promote the application of high-strength steel in steel–concrete composite structures, to further control and optimize the section size of steel–concrete structures, to improve the space utilization rate, and to reduce the amount of steel. However, research on high-strength steel in steel–concrete structures is rarely published, and the relevant specifications are still blank, which limits the use of high-strength steel in steel–concrete composite structures to a certain extent.

For a period of time, due to gaps in the Chinese, European, and American specifications [8,9,10], research on the application of high-strength steel in structures has been carried out continuously. Wang studied the overall buckling behavior of a Q420-Q960 H-type specimen under axial load, compared the specifications of various countries, and proposed a revised formula [11]. High-strength concrete-filled steel box columns under high axial compression were investigated to provide reasonable design suggestions by comparing national specifications [12]. The results of [13,14] regarding the application of high-strength concrete and high-strength steel bars validate their excellent seismic performance. Meanwhile, scholars [15,16,17,18,19,20,21] continue to carry out research on steel-reinforced concrete structures and have achieved remarkable research results in different areas. For example, research has been carried out on the seismic performance of section-steel high-strength concrete, section-steel recycled concrete, steel tubular steel concrete, steel-reinforced concrete structures with different hoop forms, and SRHC structures with different steel-section forms. Some scholars have successfully applied high-strength steel to steel–concrete composite structures, and the results show that high-strength steel–concrete composite structures exhibit good bearing capacity [22,23,24]. So far, there have been no systematic research reports on the seismic performance of built-in Q690 high-strength-steel-reinforced concrete columns.

In the seismic design of high-rise and super-high-rise building structures, the cross-section size is often largely due to the influence of the axial compression ratio limit. At this time, the bottom column is inevitably designed as a short column with a small shear span ratio. Brittle shear failure often occurs when earthquakes occur. Therefore, the research is carried out on short columns with a relatively small shear ratio. Thus, in this paper, 11 steel-reinforced concrete columns with built-in Q690 strength steel and 5 steel-reinforced concrete columns with built-in Q235 strength steel were designed for low-cycle loading tests. Considering the effects of steel strength grade, shear span ratio, axial compression ratio, stirrup ratio, and steel content on the seismic performance of steel-reinforced concrete columns, the influence laws of the energy dissipation capacity, ductility, and bearing capacity attenuation of built-in high-strength steel-section steel–concrete columns were explored. The applicability of the specification was verified, and a reference is provided for the application of the built-in Q690 high-strength steel composite structure and subsequent supplements and revisions of the specification.

## 2. Experimental Investigation

### 2.1. Test Specimens

**Figure 1:**
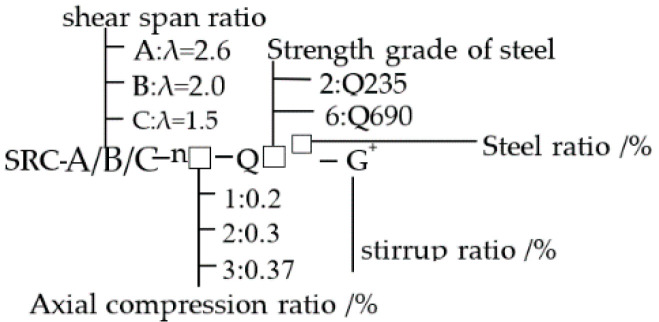
Labeling rule of specimens.

**Figure 2:**
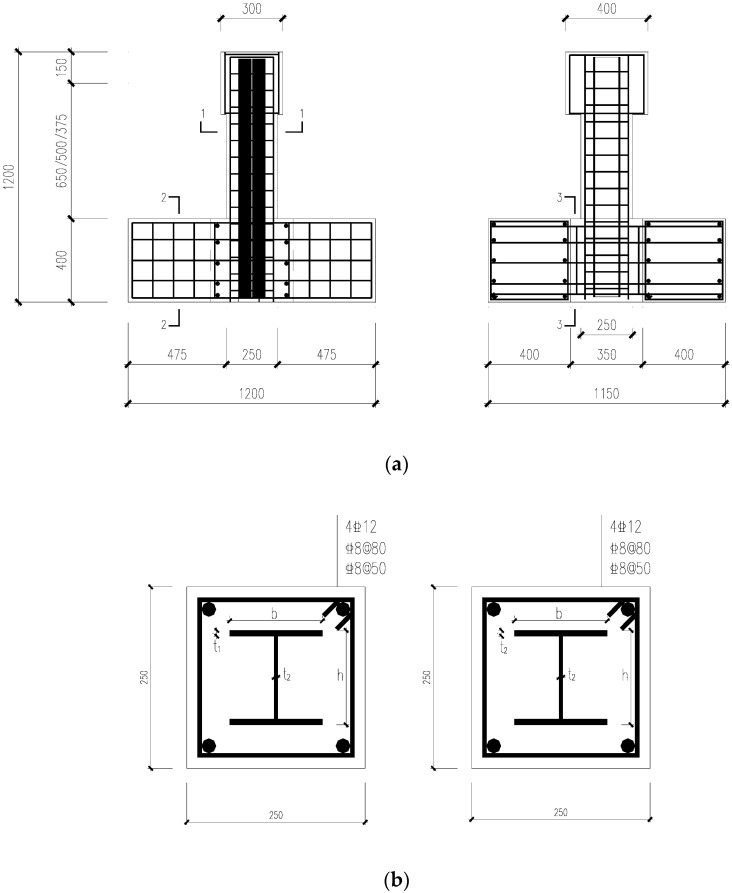

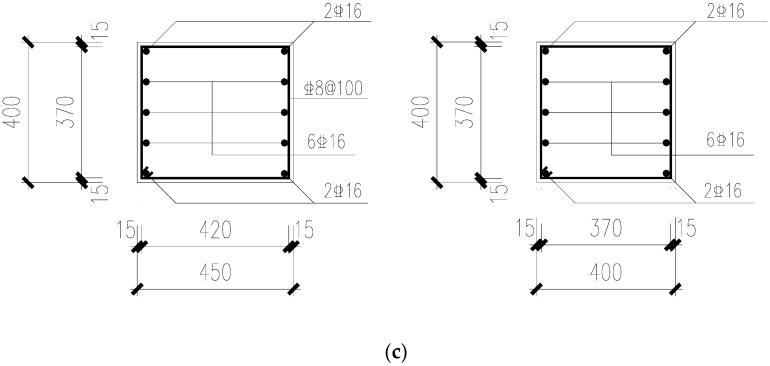
Cross-section and reinforcement of specimens (dimensions are presented in millimeters). (**a**) Specimen Elevation View (**b**) Specimen 1-1 Sectional View (**c**) Specimen 2-2; 3-3 Sectional View.

The chosen varying parameters for the tests are the (i) steel grade, (ii) shear span ratio, (iii) axial compression ratio, (iv) stirrup ratio, and (v) steel content. The test specimens were all made of C50 commercial concrete, and the detailed parameters of each specimen are shown in Table 1. The section size and reinforcement of the specimens are shown in Figure 1 and Figure 2.

### 2.2. Material Properties

**Figure 3:**
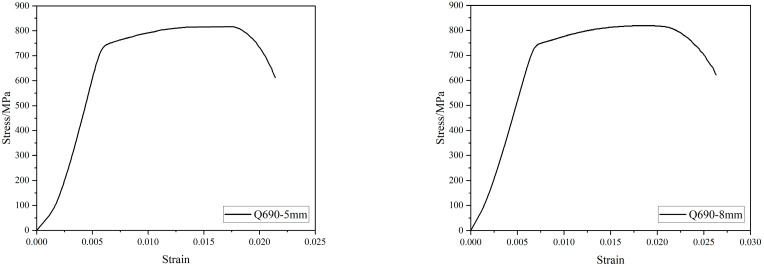

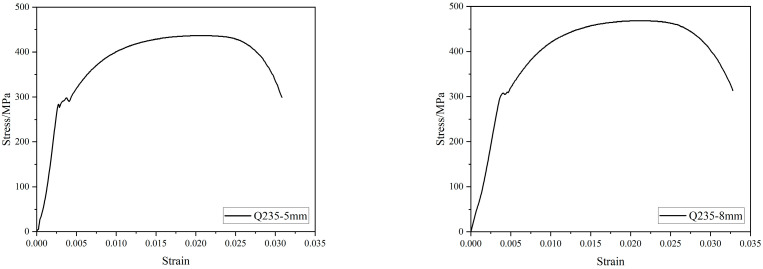
Curves of the material stress-strain.

The section steel was welded with grades Q235 and Q690 5 mm and 8 mm steel plates, respectively. We cut 3 standard parts along the length direction in the middle of the steel plate. For rebar, we adopted HRB400-grade steel bar. According to the relevant standard “Metallic Materials Tensile Test” (GB/T 228.1-2010) [25], the mechanical properties of the section steel and the steel bars were tested, as shown in Table 2. The stress-strain curve of the section steel was shown in Figure 3.

According to the relevant standard “Standard for Test Methods of Concrete Structures” (GB/T 50152-2012) [26], the mechanical properties of the concrete were tested. Nine concrete cubes (150 mm × 150 mm × 150 mm) were maintained under the same conditions as the specimens for 28 days, and the cubic compressive strength of the concrete test block was 55.6 MPa.

### 2.3. Test Setup and Procedure

For the test, we adopted cantilever beam loading. The test was carried out in the civil engineering structure pipe of Northeast Forestry University. First, a 2000 kN hydraulic jack was used to apply a constant vertical load on the top of the column. A horizontal low-cycle repetitive load on the top of the column was applied by a 1000 kN electro-hydraulic servo actuator (MTS) (Mechanical Testing & Simulation, Eden City, MN, USA).

**Figure 4:**
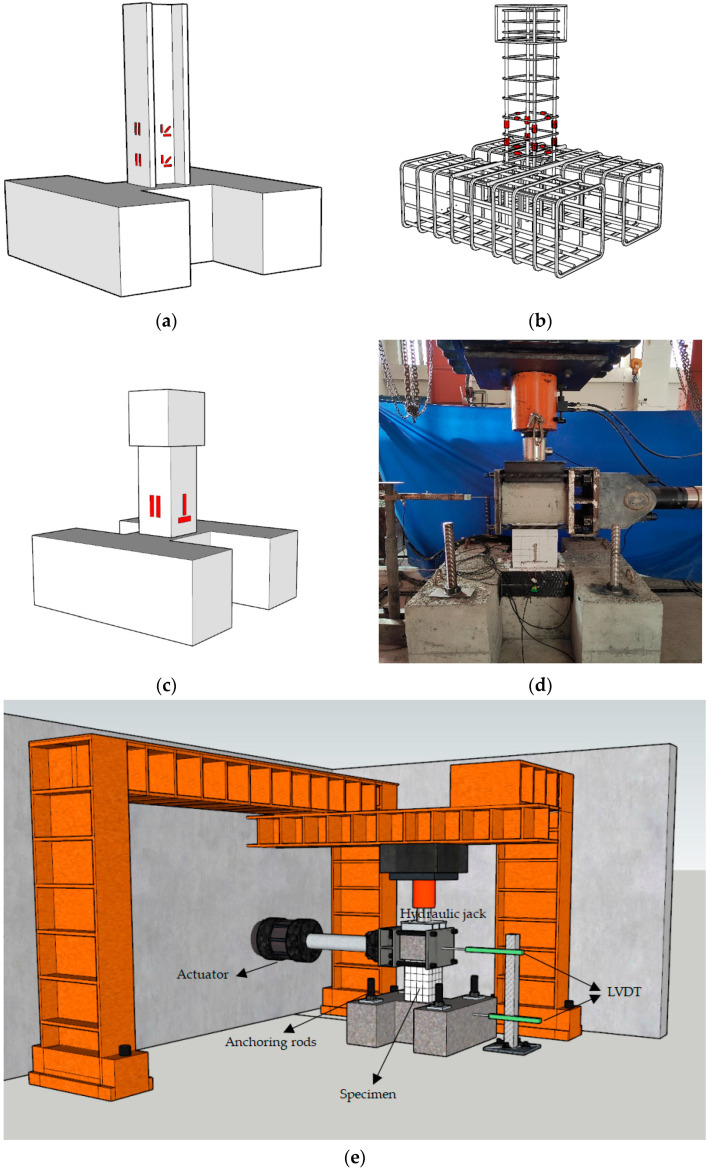
Specimen measuring points and loading equipment diagrams. (**a**) Specimens with steel; (**b**) Reinforcement framework; (**c**) Concrete; (**d**) Diagram of the test device; (**e**) Device diagram.

According to “Specification for seismic test of buildings” JGJ/T101-2015 [27], displacement-controlled loading was adopted in the test. Before the test started, preloading was carried out with an increment of 10 kN, and a cycle was started first to check whether the instrumentation was working normally and whether the connection between the loading plate and the top of the column was firm. For the horizontal load, we adopted the loading method of displacement control. The displacement angles of the first three stages of loading were 0.25%, 0.50%, and 0.75%, and each stage was cycled once; after that, the displacement angles of each stage were 1.0%, 1.5%, and 2.0%, with three cycles per level. Loading ended when one of the following occurred: (1) the column could no longer bear the axial pressure; or (2) the horizontal load dropped below 85% of the maximum load. A diagram of the test device is shown in Figure 4.

### 2.4. Data Acquisition

The main measurement contents were the horizontal load and horizontal displacement at the loading point at the top of the column, section steel strain, longitudinal reinforcement strain, stirrup strain, and concrete strain.

Firstly, the steel strain gauge is pasted at 50 mm and 150 mm away from the bottom of the column. The steel bar strain gauge is pasted between two stirrups at the bottom of the column. Finally, the concrete strain gauge is pasted at 50 mm from the bottom of the column before loading. The detailed measuring point layout is shown in Figure 4.

During the test, the strain of each part of the test piece during the test loading process is monitored in real time through the strain gauge data. The strain data are collected and recorded in real time by JM3813 (Jingming Technology Co., Ltd., Yangzhou, China) static resistance strain acquisition instrument. Horizontal load is measured by load sensors in MTS actuators. Horizontal displacement of specimen is connected with JM3813 static resistance strain acquisition instrument by LVDT (Liyang Instrument Factory, Liyang, China) and real-time acquisition by computer.

## 3. Specimen Failure Characteristics

The failure modes of the specimens under the composite stress of compression, bending, and shearing, mainly included shear baroclinic failure, shear bond failure, and bending failure. The load–displacement eigenvalues and failure modes of each specimen under different stress states are shown in Table 3.

### 3.1. Bending Failure

**Figure 5:**
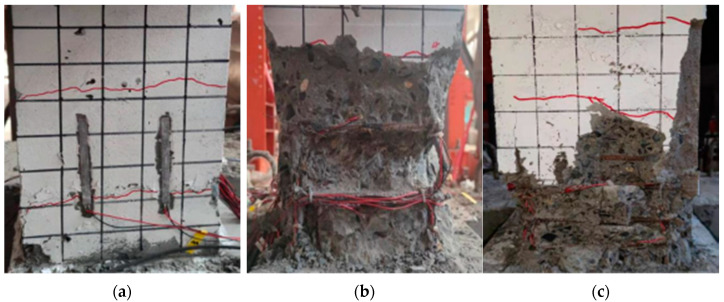
Bending Failure. (**a**) SRC-A-n_1_-Q2; (**b**) SRC-A-n_1_-Q6; (**c**) SRC-A-n_3_-Q6-G^+^.

When the shear span was large (λ = 2.6), the specimens mainly suffered bending failure. In the initial stage of loading, horizontal bending cracks appeared continuously on both sides of the root of the specimen. As the loading progressed, the bending cracks extended to the front and gradually connected. When the specimens were damaged, the compressive stress of the concrete in the compression zone reached its ultimate compressive strength, and the concrete was broken; then, the longitudinal reinforcement buckled, and the profiled steel flange and even the web in the compression zone gradually yielded. The horizontal bearing capacity of the specimens was small, the failure process was relatively slow, and the ductility was good. The failure mode of the specimen was shown in Figure 5.

### 3.2. Shear Bond Failure

**Figure 6:**
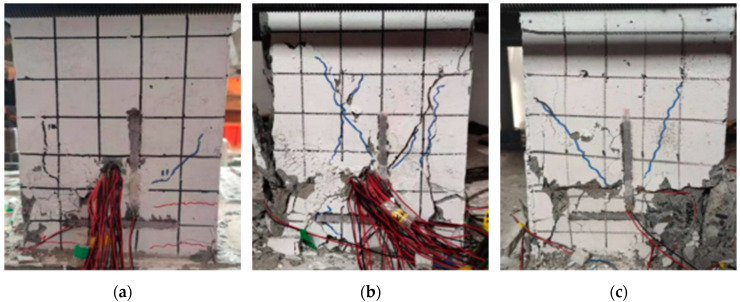
Shear bond failure. (**a**) SRC-B-n_1_-Q2; (**b**) SRC-B-n_3_-Q6; (**c**) SRC-B-n_2_-Q6.

When the shear span ratio was moderate (λ = 2.0), shear bond failure of the specimens mainly occurred. At the initial stage of loading, the specimens were basically in the elastic stage; as the loading progressed, the specimens began to exhibit horizontal cracks and diagonal cracks, but the development was relatively slow. When loaded into a certain cycle, longitudinal bond cracks distributed along the height of the column appeared in the concrete outside the steel compression flange and developed rapidly; eventually, a major longitudinal bond-splitting crack formed. As the number of cycles continued to increase, the concrete protective layer continued to peel off, the stiffness of the specimen decreased, and the horizontal load decreased sharply. This eventually led to failure of the specimens. The horizontal bearing capacity of the specimens was relatively small, the failure process was short, and the ductility was poor. The failure mode of the specimen was shown in Figure 6.

### 3.3. Shear Baroclinal Failure

**Figure 7:**
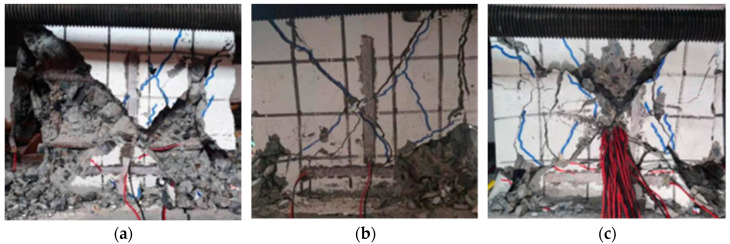
Shear baroclinal failure. (**a**) SRC-C-n_2_-Q6; (**b**) SRC-C-n_1_-Q6; (**c**) SRC-C-n_1_-Q2.

When the shear span was small (λ = 1.5), the specimens mainly suffered from shear baroclinic failure. At the initial stage of loading, the horizontal load was small, and the specimens were basically in the elastic stage. As the loading continued, oblique cracks appeared near the axis of the abdomen of the specimens, and at the same time, a few vertical bonding cracks appeared on the outside of the section-steel compression flange; as the load increased, the bonding cracks and the oblique cracks continued to develop. However, the development of the inclined crack was relatively rapid. With the development of the crack diagonally and crosswise under the action of the cyclic reciprocating load, the specimen was divided into several prisms, causing the concrete to withdraw from the work and the specimens to be damaged. The horizontal bearing capacity of the specimens was relatively large, the failure process was relatively rapid, and the ductility was poor. The failure mode of the specimen was shown in Figure 7.

## 4. Test Results and Analysis

### 4.1. Hysteresis Curves

**Figure 8:**
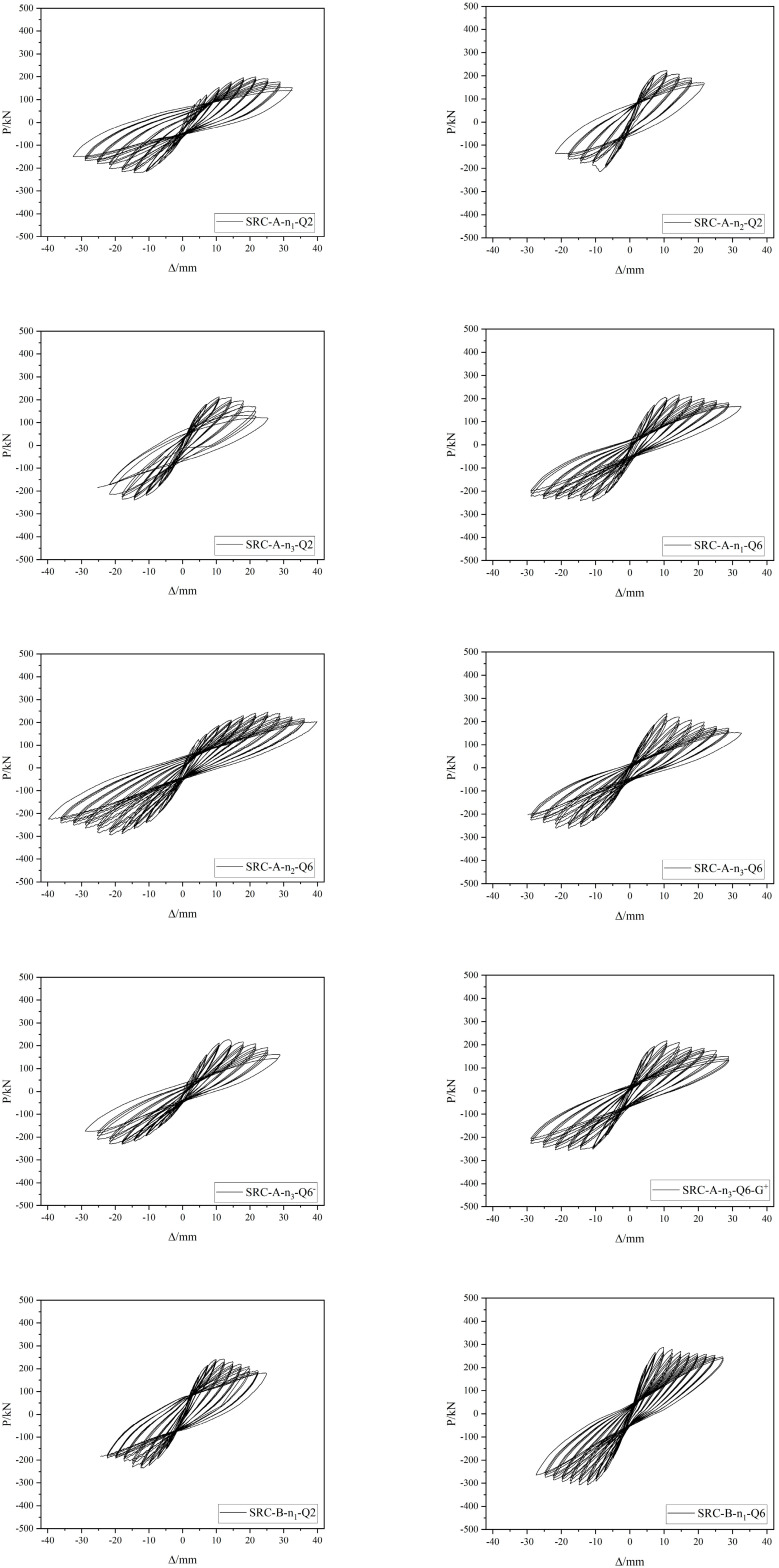

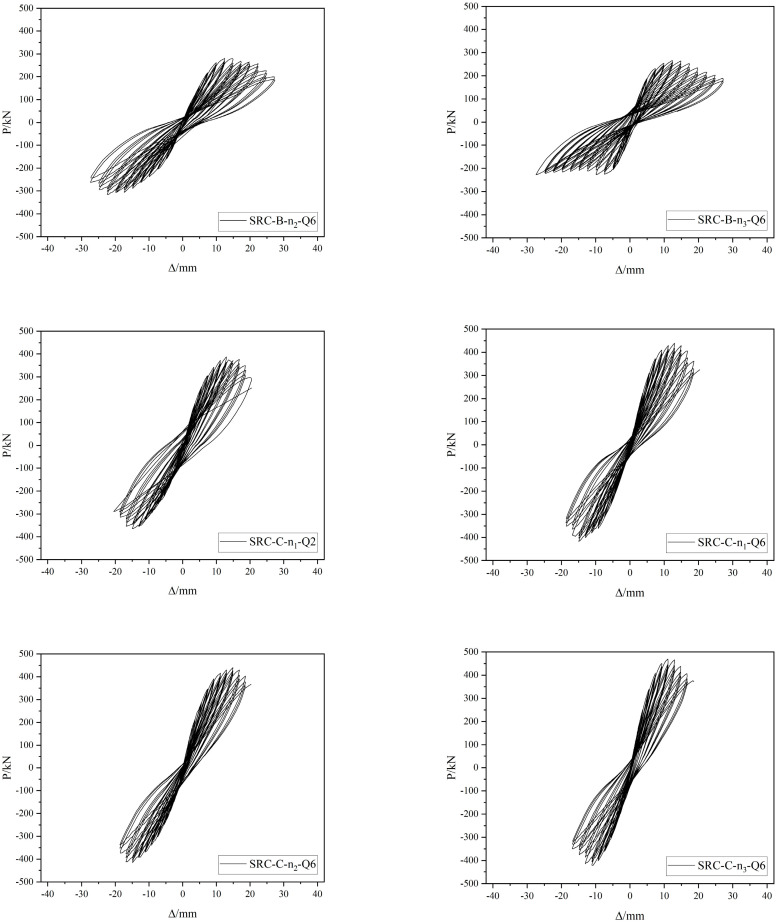
Hysteresis loops of the specimens.

The hysteresis curves of the SRC columns are shown in Figure 8, and the following can be seen in the figure:

1. When the horizontal load is less than the cracking load, the P–Δ curve is straight, and the specimen is basically in the elastic stage; as the load increases, the P–Δ curve gradually deviates from the straight line, the deformation accelerates, residual deformation occurs when unloading, and the specimen enters the elastic–plastic stage. When the load reaches the yield load, the slope of the loading curve decreases with increasing displacement, and the magnitude of the decrease gradually accelerates. In the three cycles of the same displacement control loading stage, the slope and maximum load of the latter loading curve are smaller than those of the previous one, and with an increasing number of cycles, the stiffness and bearing capacity attenuate significantly. After reaching the ultimate load, the unloading curve is steep, the deformation recovery is small, and the displacement lags significantly.
2. Under the same conditions for the other parameters, the specimens with a large shear span ratio bear a less ultimate horizontal load, the horizontal load decays slowly, the hysteresis loop is relatively full, the number of cycles experienced during a failure is large, and the horizontal displacement is large. As the shear span ratio decreases, the hysteresis curve of the specimen shrinks, and the specimen should be strengthened with structural measures to achieve better performance.
3. When the built-in Q690 steel is damaged, compared with the built-in Q235 steel, the ultimate horizontal bearing capacity is greater and the hysteresis loop is slightly narrower, but the section steel bears most of the horizontal shear and vertical loads in the later stage of loading. Therefore, the stability and ultimate deformation of the built-in Q690 steel specimen are relatively large.
4. The ultimate load of the specimen with the larger axial pressure is larger, but after reaching the ultimate load, the load decays faster and the overall damage is faster. However, when the specimen with the built-in Q690 steel is damaged, it will bear more cyclic loads, and the load decays more slowly.
5. The specimens with a larger steel content and hoop ratio have fuller hysteresis loops, more loading cycles, stronger deformation capacity at failure, larger limit displacement, and stronger energy dissipation capacity.

### 4.2. Skeleton Curves

**Figure 9:**
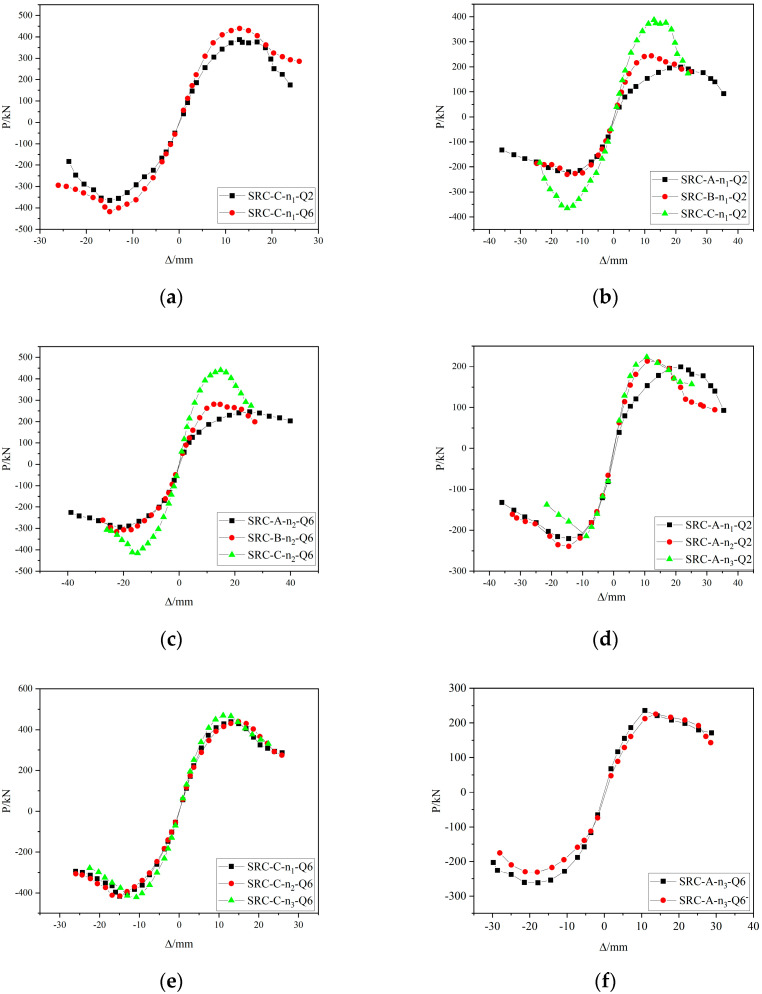

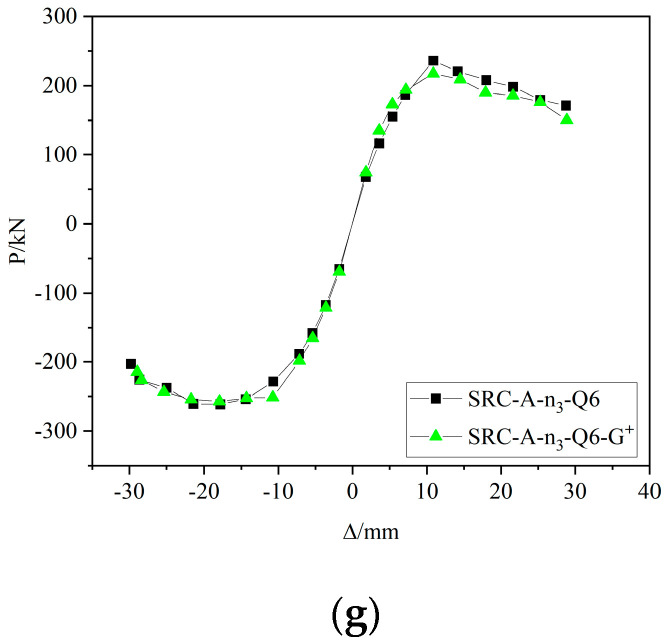
Skeleton curves of the specimens. (**a**) steel strength; (**b**) shear span ratio (Q235); (**c**) shear span ratio (Q690); (**d**) axial compression ratio (Q235); (**e**) axial compression ratio (Q690); (**f**) steel content; (**g**) stirrup ratio.

The influence of the design parameters on the skeleton curves of the specimens is shown in Figure 9.

1. The stress process of each specimen can be divided into four stages: elasticity, concrete cracking elastoplasticity, section-steel yielding, and failure. Compared to ordinary SRC frame columns, due to the built-in high-strength steel, the ultimate load of the specimen is increased, and it provides better ductility and deformation capacity. The descending section of the skeleton curve is relatively gentle, the load decays slowly, and the plastic deformation ability is strong. The influence of steel strength on the skeleton curve is shown in Figure 9a.
2. The influence of the shear span ratio of the specimen on its stress is mainly reflected in the failure form and the ultimate bearing capacity. Both ordinary SRC frame columns and HSRC frame columns show the following: With an increasing shear span ratio, the specimens successively suffered a shear oblique failure, shear bond failure, and bending failure, and the bearing capacity in turn decreased. The ascending and descending sections of the skeleton curve tend to be gentle, the limit displacement increases gradually, and the ductility increases. The effect of the shear span ratio on the skeleton curve is shown in Figure 9b,c.
3. With an increasing axial compression ratio, the ultimate bearing capacity of the specimen increases, and the decay speed becomes faster after reaching the ultimate load, resulting in a steeper descending section of the skeleton curve of the specimen, poor plasticity, and reduced ductility. However, the decay rate of the HSRC frame column is slower than that of the ordinary SRC frame column after reaching the ultimate load for different axial compression ratios, which reflects better plastic deformation capacity. The effect of the axial compression ratio on the skeleton curve is shown in Figure 9d,e.
4. The influence of SRC-A-n_3_-Q6^−^ with a small steel content and SRC-A-n_3_-Q6-G^+^ with a larger hoop ratio on the skeleton curves of the specimens is shown in Figure 9f,g. With increasing hoop ratio and steel content, the ultimate deformation capacity of the specimen increases, and the descending section of the skeleton curve tends to be gentle.

### 4.3. Displacement Ductility

**Figure 10:**
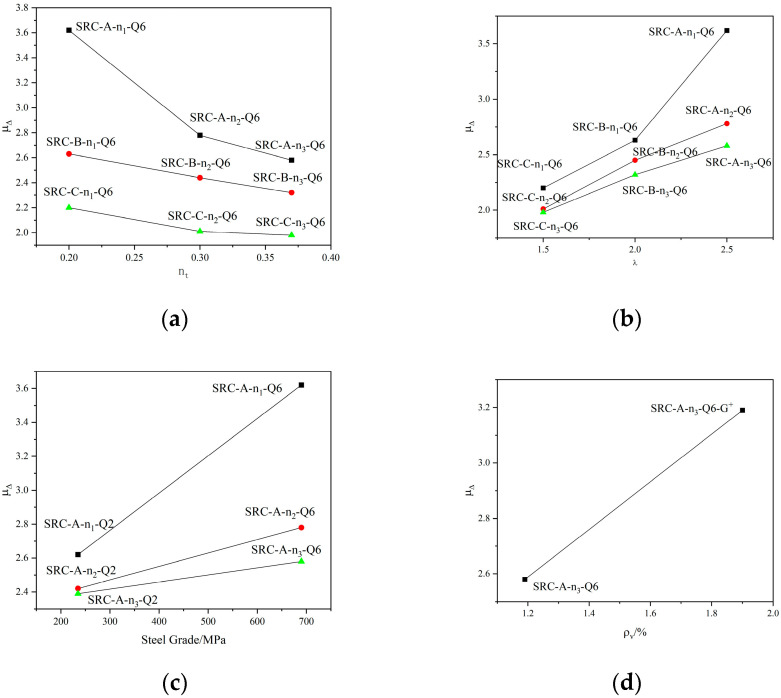

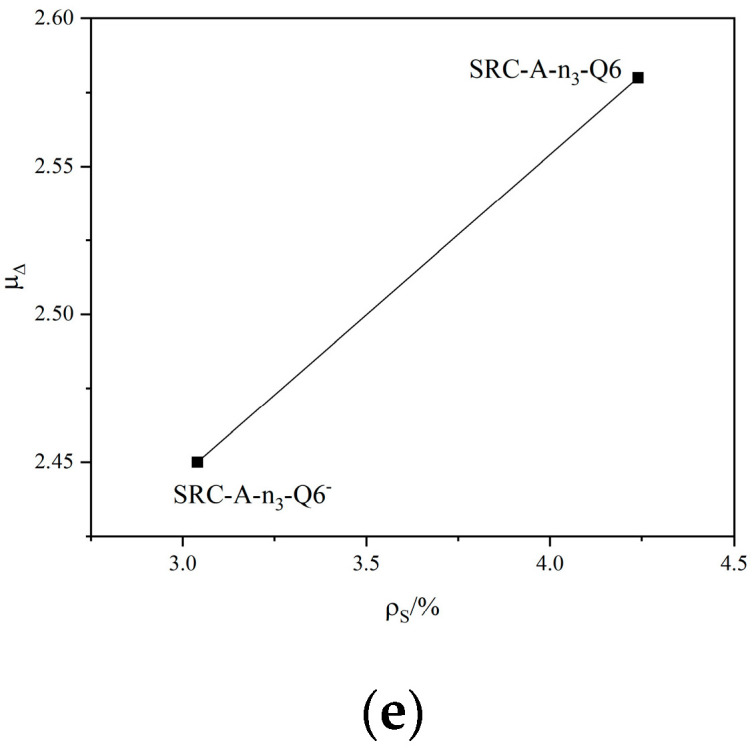
Displacement ductility coefficients of the specimens. (**a**) axial compression ratio; (**b**) shear span ratio; (**c**) Steel strength grade; (**d**) stirrup ratio; (**e**) steel ratio.

Ductility is an important index used to characterize the deformation capacity, usually expressed by the displacement ductility coefficient μ_Δ_, and the equivalent energy method is used to determine the yield displacement Δ_y_ of the specimen. From the skeleton curve, the horizontal displacement corresponding to 0.85 pmax was determined as the failure displacement Δ_u_ of the specimen. Then, the displacement ductility coefficient is defined as μ_Δ_ = Δ_u_/Δ_y_. The displacement ductility coefficients of the specimens calculated in this manner are shown in Table 4, and the influence of the design test parameters on the displacement ductility coefficients of the specimens is shown in Figure 10. The following can be seen in Table 4 and Figure 10:

1. The displacement ductility coefficient of the specimen decreases with an increasing axial compression ratio. For specimens SRC-A-n_1_-Q6, SRC-A-n_2_-Q6, and SRC-A-n_3_-Q6, the displacement ductility coefficients are 3.62, 2.78, and 2.58. The displacement ductility coefficient decreases sequentially; as the axial pressure ratio increases from 0.2 to 0.37, the displacement ductility coefficient of the specimen decreases by 28.73%. The increase in the axial compression ratio increases the principal compressive stress and principal compressive strain of the specimen, which weakens the ultimate deformation capacity of the concrete and reduces the ductility of the specimen. Moreover, the increase in the axial pressure increases the p–Δ effect of the specimen, resulting in an increase in the secondary additional bending moment and deformation, and the ductility of the specimen is weakened. The influence of the axial compression ratio on the displacement ductility coefficients of the specimens is shown in Figure 10a.
2. The displacement ductility coefficient of the specimens increases with an increasing shear span ratio; for the specimens SRC-A-n_1_-Q6 and SRC-A-n_1_-Q2 with a larger shear span ratio, the displacement ductility coefficients are 3.62 and 2.62, respectively. For the specimens with a small shear span ratio, SRC-C-n_1_-Q6 and SRC-C-n_1_-Q2, the displacement ductility coefficients are 2.20 and 1.89, respectively. For specimens SRC-A-n_1_-Q6 and SRC-C-n_1_-Q6, the shear span ratio is reduced from 2.5 to 1.5. The displacement ductility coefficients of the specimens decreased by 39.23%. It can be seen that the effect of the shear span ratio on the displacement ductility coefficients of the specimens follows a positive correlation. Moreover, specimens with small shear span ratios often suffer from brittle shear–oblique failures, so it is advisable to avoid designing small shear-span-ratio specimens in engineering design. The effect of the shear span ratio on the displacement ductility coefficients of the specimens is shown in Figure 10b.
3. The displacement ductility coefficient of the specimens increases with increasing steel strength grade. For specimen SRC-A-n_1_-Q6 with built-in Q690 steel and specimen SRC-A-n_1_-Q2 with built-in Q235 steel, the displacement ductility coefficients are 3.62 and 2.62, respectively. From the built-in Q235 steel to the built-in Q690 steel, the displacement ductility coefficient increases by 38.17%. It can be seen that the built-in high-strength steel can provide a relatively good improvement in the displacement ductility coefficient of the specimens. The influence of steel strength on the displacement ductility coefficient is mainly reflected in the fact that the steel strength can bear the horizontal shear force well and provide good deformation ability, which causes the displacement ductility coefficient of the specimen to increase. The influence of steel strength on the displacement ductility coefficients of the specimens is shown in Figure 10c.
4. With increasing hoop ratio and steel content, the displacement ductility coefficients of the specimens increase. The hoop ratio of specimens SRC-A-n_3_-Q6 (p_v_ = 1.19%) and SRC-A-n_3_-Q6-G^+^ (p_v_ = 1.90%) increased. The corresponding displacement ductility coefficients are 2.58 and 3.19, respectively. It can be seen that as the hoop ratio increases, the displacement ductility coefficient increases. The displacement ductility coefficients of specimen SRC-A-n_3_-Q6^−^ (p_s_ = 3.04%) with a small steel content and specimen SRC-A-n_3_-Q6 (p_s_ = 4.24%) with a large steel content are 2.45 and 2.58, respectively. It can be seen that the displacement ductility coefficient of the specimen increases with increasing steel content. The increase in the hoop ratio and the steel content can effectively restrain the deformation of the concrete, bring the concrete to a state of multi-directional compression, effectively improve the ultimate deformation capacity of the concrete, and have a positive impact on the displacement ductility coefficient of the specimen. The influence of the hoop ratio and steel content on the displacement ductility coefficients of the specimens is shown in Figure 10d,e.

### 4.4. Energy Dissipation Capacity

**Figure 11:**
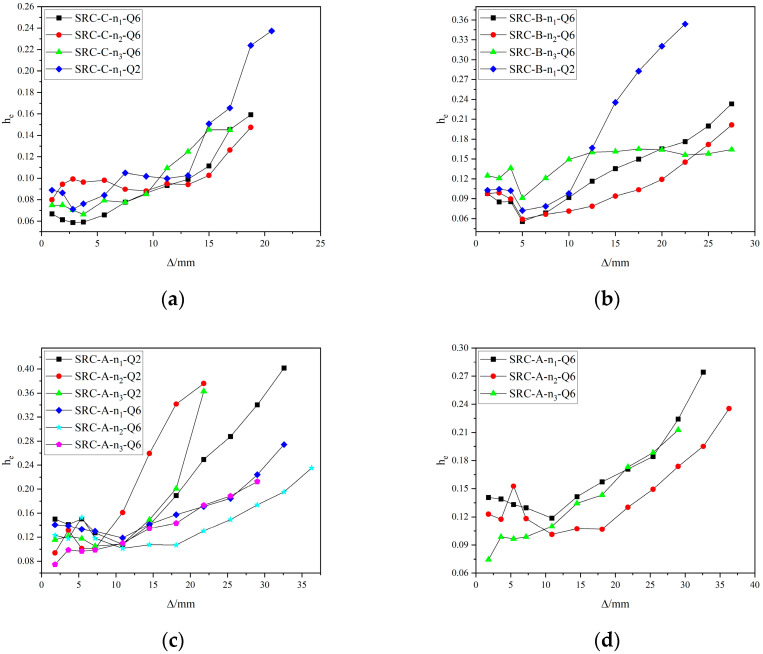

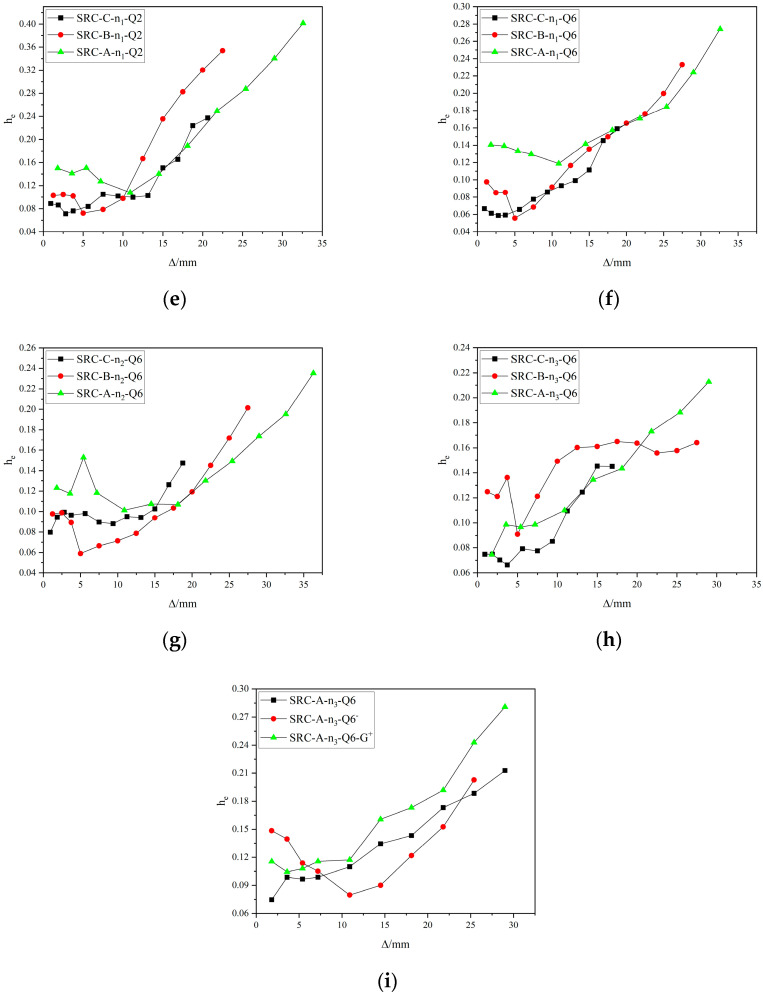
Influence of design parameters on the equivalent viscous damping coefficients of specimens. (**a**) λ = 1.5; (**b**) λ = 2.0; (**c**) λ = 2.6; (**d**) λ = 2.6 (Q690); (**e**) *n* = 0.2 (Q235); (**f**) *n* = 0.2 (Q690); (**g**) *n* = 0.3 (Q690); (**h**) *n* = 0.37 (Q690); (**i**) stirrup ratio and steel ratio.

The energy dissipation performance of structural members is an important basis for evaluating their seismic performance. If the structural members have a good energy dissipation performance under a certain strength guarantee, a large part of the energy can be dissipated during the earthquake process, so that the structure will not be seriously damaged. The energy dissipation performance is usually described by the equivalent viscosity coefficient h_e_, where h_e_ = A/2πFΔ. In this formula, A is the area of a hysteresis loop and FΔ is the average value of the product of the maximum horizontal load and the maximum horizontal displacement of the upper and lower parts of the hysteresis loop. The larger the h_e_ value, the stronger the energy dissipation capacity of the component. Figure 11 shows the relationship between h_e_ and the deformation of some specimens in the last cycle under different displacements.

1. The equivalent viscous damping coefficients of the specimens increase with increasing displacement, and the specimens’ h_e_ values show a sudden change under the same displacement cycle with different cycles. In the later stage of loading, the h_e_ values of the specimens can maintain a certain growth, but the growth rate is reduced. This shows that in the later stage of loading, although the concrete protective layer is continuously crushed and peeled off, the steel and stirrup-constrained concrete in the core area can still bear the horizontal load and dissipate the seismic energy well. It has a good energy dissipation capacity, and it is also beneficial for resisting secondary aftershocks after major earthquakes.
2. With an increasing axial compression ratio of the specimens, their equivalent viscous damping coefficients gradually decrease, indicating that an increase in the axial compression ratio is not good for the seismic performance of the specimens. The ultimate energy consumption index of specimen SRC-C-n_1_-Q6 (h_e_ = 0.16) is greater than that of specimen SRC-C-n_3_-Q6 (h_e_ = 0.14). The limit energy consumption index of specimen SRC-A-n_1_-Q6 (h_e_ = 0.27) is greater than that of specimen SRC-A-n_3_-Q6 (h_e_ = 0.21). The influence of the axial compression ratio on the equivalent viscous damping coefficients of the specimens is shown in Figure 11a–d.
3. With an increasing shear span ratio of the specimens, the equivalent viscous damping coefficients of the specimens increase gradually, indicating that an increase in the shear span ratio can improve the seismic performance of the specimens. The limit energy consumption index of specimen SRC-C-n_2_-Q6 (h_e_ = 0.15) is less than that of specimen SRC-A-n_2_-Q6 (h_e_ = 0.24). The limit energy consumption index of specimen SRC-C-n_1_-Q2 (h_e_ = 0.24) is less than that of specimen SRC-A-n_1_-Q2 (h_e_ = 0.43). The effect of the shear span ratio on the equivalent viscous damping coefficients of the specimens is shown in Figure 11e–h.
4. With increasing steel strength grade from Q235 to Q690, the equivalent viscous damping coefficient of the specimen gradually decreases. The limit energy consumption index of specimen SRC-A-n_2_-Q6 (h_e_ = 0.24) is less than that of specimen SRC-A-n_2_-Q2 (h_e_ = 0.40). The limit energy consumption index of specimen SRC-B-n_1_-Q6 (h_e_ = 0.23) is less than that of specimen SRC-B-n_1_-Q2 (h_e_ = 0.35). The effect of steel strength on the equivalent viscous damping coefficients of the specimens is shown in Figure 11a–c.
5. An increase in the hoop ratio and steel content can effectively constrain the concrete in the core area, improve the plastic deformation ability of the specimen, and improve the seismic performance of the specimen. The limit energy consumption index of specimen SRC-A-n_3_-Q6 (h_e_ = 0.21) is greater than that of specimen SRC-A-n_3_-Q6^−^ (h_e_ = 0.20). The limit energy consumption index of specimen SRC-A-n_3_-Q6 (h_e_ = 0.21) is less than that of specimen SRC-A-n_3_-Q6-G^+^ (h_e_ = 0.28). The effect of hoop ratio and steel content on the equivalent viscous damping coefficients of the specimens is shown in Figure 11i.

## 5. Conclusions

1. The SRC frame columns with built-in high-strength steel performed well with regard to the various seismic performance indicators; the SRC columns equipped with Q690 steel have obvious advantages in terms of bearing capacity and displacement ductility, and their energy dissipation capacity is lower than that of ordinary SRC frame columns.
2. Under the action of low-cycle repeated loads, with an increasing axial compression ratio, the horizontal bearing capacity of the SRC frame column increases, and the energy dissipation and displacement ductility performance decrease, but the decrease for the SRC column configured with Q690 steel is slightly slower.
3. With an increasing shear span ratio, the horizontal bearing capacity of the SRC frame column decreases, the load decays rapidly, and the energy dissipation and displacement ductility gradually increase. High-strength steel behaves in the same way as ordinary steel.
4. With increasing hoop ratio and steel content, the horizontal bearing capacity of the built-in Q690 steel frame column is improved, the load decay rate slows down, and the displacement ductility performance and energy dissipation capacity are improved.

## Figures and Tables

**Table 1:** Details of specimens.

Specimen Designation	*λ*	*n*	p_s_ (%)	p_v_ (%)	Steel Grade
SRC-A-n_1_-Q6	2.6	0.2	4.24	1.19	Q690
SRC-A-n_2_-Q6	2.6	0.3	4.24	1.19	Q690
SRC-A-n_3_-Q6	2.6	0.37	4.24	1.19	Q690
SRC-B-n_1_-Q6	2	0.2	4.24	1.19	Q690
SRC-B-n_2_-Q6	2	0.3	4.24	1.19	Q690
SRC-B-n_3_-Q6	2	0.37	4.24	1.19	Q690
SRC-C-n_1_-Q6	1.5	0.2	4.24	1.19	Q690
SRC-C-n_2_-Q6	1.5	0.3	4.24	1.19	Q690
SRC-C-n_3_-Q6	1.5	0.37	4.24	1.19	Q690
SRC-A-n_1_-Q2	2.6	0.2	4.24	1.19	Q235
SRC-A-n_2_-Q2	2.6	0.3	4.24	1.19	Q235
SRC-A-n_3_-Q2	2.6	0.37	4.24	1.19	Q235
SRC-B-n_1_-Q2	2	0.2	4.24	1.19	Q235
SRC-C-n_1_-Q2	1.5	0.2	4.24	1.19	Q235
SRC-A-n_3_-Q6^−^	2.6	0.37	3.04	1.19	Q690
SRC-A-n_3_-Q6-G^+^	2.6	0.37	4.24	1.90	Q690

**Table 2:** Mechanical properties of steel.

Grade	Specification	f_y_/MPa	f_u_/MPa	Elongation Ratio/%
Q235	5 mm	277.15	437.28	32.1
Q235	8 mm	304.84	468.73	30.4
Q690	5 mm	696.83	819.80	19.6
Q690	8 mm	695.48	818.22	21.4
HRB400	8 mm	457.6	611.2	26.8
HRB400	12 mm	428.7	578.6	28.7

**Table 3:** Load characteristic values and failure mode of each specimen under different stress states.

Specimen Designation	p_cr_/kN	Δ_cr_/mm	p_y_/kN	Δ_y_/mm	P_m_/kN	Δ_m_/mm	p_u_/kN	Δ_u_/mm	Destruction Form
SRC-A-n_1_-Q6	108.55	4.29	193.08	9.01	217.58	14.42	182.25	28.89	Bending failure
SRC-A-n_2_-Q6	123.69	5.33	206.95	13.69	245.82	25.35	203.54	39.89	Bending failure
SRC-A-n_3_-Q6	142.44	5.45	226.09	10.49	261.35	21.44	225.75	28.65	Bending failure
SRC-B-n_1_-Q6	125.85	2.59	272.59	8.12	288.27	9.89	247.12	27.06	Shear Bond failure
SRC-B-n_2_-Q6	122.43	2.44	253.04	9.42	281.09	12.44	227.47	24.80	Shear Bond failure
SRC-B-n_3_-Q6	131.09	3.12	235.37	7.81	266.34	12.38	215.43	22.01	Shear Bond failure
SRC-C-n_1_-Q6	112.16	1.83	392.62	8.39	417.60	14.95	351.44	18.54	Shear Baroclinic failure
SRC-C-n_2_-Q6	116.81	1.73	390.68	9.22	440.04	14.92	366.38	20.31	Shear Baroclinic failure
SRC-C-n_3_-Q6	130.47	1.86	419.68	7.87	469.34	11.06	406.69	16.62	Shear Baroclinic failure
SRC-A-n_1_-Q2	98.10	5.66	169.92	7.87	199.21	21.58	153.12	31.23	Bending failure
SRC-A-n_2_-Q2	154.57	4.24	187.87	6.90	223.29	10.64	170.79	19.46	Bending failure
SRC-A-n_3_-Q2	154.58	5.36	186.79	7.76	213.59	10.83	171.10	19.31	Bending failure
SRC-B-n_1_-Q2	121.84	3.12	216.59	7.46	243.42	12.17	210.53	19.54	Shear Bond failure
SRC-C-n_1_-Q2	81.42	1.62	342.44	9.24	372.11	11.19	310.19	18.45	Shear Baroclinic failure
SRC-A-n_3_-Q6^−^	132.44	5.71	204.07	10.27	225.69	13.81	192.27	25.26	Bending failure
SRC-A-n_3_-Q6-G^+^	184.24	6.18	227.19	9.11	257.17	17.93	214.51	28.94	Bending failure

**Table 4:** The equivalent viscous damping coefficients and ductility factors of the specimens.

Specimen Designation	h_e_	μ_Δ_	Specimen Designation	h_e_	μ_Δ_
SRC-A-n_1_-Q6	0.27	3.62	SRC-C-n_3_-Q6	0.14	1.98
SRC-A-n_2_-Q6	0.24	2.78	SRC-A-n_1_-Q2	0.43	2.62
SRC-A-n_3_-Q6	0.21	2.58	SRC-A-n_2_-Q2	0.40	2.42
SRC-B-n_1_-Q6	0.23	2.63	SRC-A-n_3_-Q2	0.37	2.39
SRC-B-n_2_-Q6	0.20	2.45	SRC-B-n_1_-Q2	0.35	2.55
SRC-B-n_3_-Q6	0.16	2.32	SRC-C-n_1_-Q2	0.24	1.89
SRC-C-n_1_-Q6	0.16	2.20	SRC-A-n_3_-Q6^−^	0.20	2.45
SRC-C-n_2_-Q6	0.15	2.01	SRC-A-n_3_-Q6-G^+^	0.28	3.19

## Data Availability

The data presented in this study are available on request from the corresponding author.

## Nomenclature

SRC	steel reinforced concrete
SRHC	steel reinforced high strength concrete
*n*	axial compression ratio
λ	shear span ratio
p_v_	stirrup ratio
p_s_	steel content
f_y_	yield strength of steel and rebar
f_u_	ultimate strength of steel and rebar
p, Δ	lateral load and corresponding displacement, respectively
p_cr_, Δ_cr_	crack load and corresponding displacement, respectively
p_y_, Δ_y_	yield load and corresponding displacement, respectively
p_m_, Δ_m_	maximum load and corresponding displacement, respectively
p_u_, Δ_u_	ultimate load and corresponding displacement, respectively
μ_Δ_	ductility factor
h_e_	equivalent viscous damping coefficient
